# Evaluating the spatial distribution of quantitative risk and hazard level of arsenic exposure in groundwater, case study of Qorveh County, Kurdistan Iran

**DOI:** 10.1186/1735-2746-10-30

**Published:** 2013-04-10

**Authors:** Touraj Nasrabadi, Niloufar Shirani Bidabadi

**Affiliations:** 1 University of Tehran, Azin Avenue, Ghods Street, Enghelab Square, #23, zip code: 1417853111, Tehran, Iran

**Keywords:** Arsenic, Groundwater, Risk, Hazard, Qorveh, Drinking water

## Abstract

Regional distribution of quantitative risk and hazard levels due to arsenic poisoning in some parts of Iran’s Kurdistan province is considered. To investigate the potential risk and hazard level regarding arsenic-contaminated drinking water and further carcinogenic and non-carcinogenic effects on villagers, thirteen wells in rural areas of Qorveh County were considered for evaluation of arsenic concentration in water. Sampling campaign was performed in August 2010 and arsenic concentration was measured via the Silver Diethyldithiocarbamate method. The highest and lowest arsenic concentration are reported in Guilaklu and Qezeljakand villages with 420 and 67 μg/L, respectively. None of thirteen water samples met the maximum contaminant level issued by USEPA and Institute of Standards and Industrial Research of Iran (10 ppb). The highest arsenic concentration and consequently risk and hazard levels belong to villages situated alongside the eastern frontiers of the county. Existence of volcanic activities within the upper Miocene and Pleistocene in this part of the study area may be addressed as the main geopogenic source of arsenic pollution. Quantitative risk values are varying from 1.49E-03 in Qezeljakand to 8.92E-03 in Guilaklu and may be interpreted as very high when compared by similar studies in Iran. Regarding non-carcinogenic effects, all thirteen water samples are considered hazardous while all calculated chronic daily intakes are greater than arsenic reference dose. Such drinking water source has the potential to impose adverse carcinogenic and non-carcinogenic effects on villagers. Accordingly, an urgent decision must be made to substitute the current drinking water source with a safer one.

## Background

Being the twentieth abundant element, arsenic is remarkably distributed within earth crust all around the world [1]. Generally, arsenic compounds may be categorized into three gaseous, organic and inorganic ones from which the latest is considered the most toxic [2]. According to the data gathered by human epidemiological studies, arsenic is classified as carcinogenic [3,4].

Increased mortality from multiple internal organ cancers (liver, kidney, lung, and bladder) and an increased incidence of skin cancer were observed in populations consuming drinking water high in inorganic arsenic; A cross-sectional study of 40,000 Taiwanese exposed to arsenic in drinking water found significant excess skin cancer prevalence by comparison to 7500 residents of Taiwan and Matsu who consumed relatively arsenic-free water [5]. A prevalence study of skin lesions was conducted in two towns in Mexico, one with 296 persons exposed to drinking water with 0.4 mg/L arsenic and a similar group with exposure at 0.005 mg/L. The more exposed group had an increased incidence of palmar keratosis, skin hyperpigmentation and hypopigmentation, and skin cancers [6]. Authors [7] found that the standard mortality ratios (SMR) and cumulative mortality rates for cancers of bladder, kidney, skin, lung and liver were significantly greater in the area where people are exposed to arsenic contaminated water when compared with the age adjusted rates for the general population of Taiwan.

Among different exposure routes, ingestion is regarded as the most effective one through which arsenic may aversely affect the human health. Drinking water is introduced as the most wide spread media through which humans are exposed to arsenic [8]. Geopogenic resources rather than anthropogenic ones are responsible for arsenic contamination of water bodies around the world [9,10]. The main natural source of metals/metalloids including arsenic in aquatic systems is considered to be weathering of soils and rocks [11]. Chronic exposure to arsenic contaminated drinking water has been detected as the main cause of skin, liver, kidney and lung cancer reports [2]. Furthermore, skin lesions including pigmentation changes, mainly on the trunk and extremities, and keratosis of the palm of the hands and soles of the feet are also the result of chronic ingestion of inorganic arsenic [1,12-14]. A great variety of researches have indicated extremely high concentrations of arsenic in water bodies of Bangladesh, India, Vietnam, China, Nepal, Argentina, Chile, Mexico, Poland, Hungary, united states and Iran [9,15-20]. More than one hundred million people have been reported to be exposed to arsenic contaminated water just in Asia at the end of the second millennium [21,22].

Quantitative risk and hazard analysis by considering parameters like chronic daily intake, intake factor, average body weight, exposure time, frequency and duration for a whole lifetime has the capability to manifest an unbiased view of the current status. A group of researchers in China [13], Cambodia [23] and northern Pakistan [24] have quantified the risk levels relevant to chronic exposure to arsenic contaminated drinking water.

The first formal report of arsenic poisoning in Iran was documented in 1986 in Kurdistan province where a villager lost her leg due to gangrene caused by consumption of arsenic contaminated water. Different studies have focused on hazards caused by high concentrations of arsenic in groundwater of Kurdistan province during recent decades and a variety of symptoms of chronic arsenic poisoning is also detected among villagers [10,19,22,25]. In the present study the Arsenic concentration in groundwater of thirteen villages in Qorveh county, Kurdistan province is measured and the potential risk and hazard level regarding arsenic-contaminated drinking water are evaluated.

### Study area

**Figure 1:**
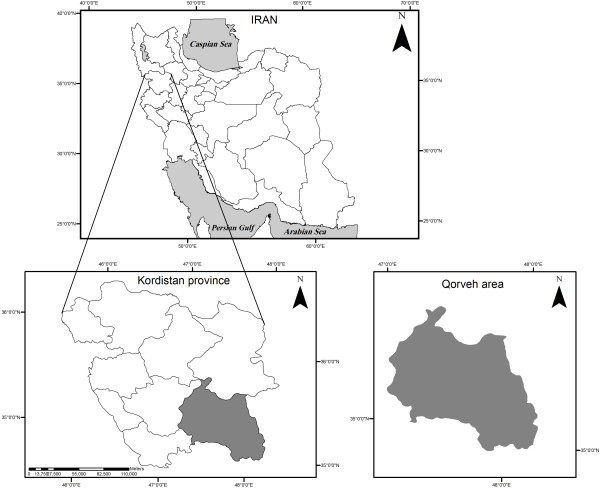
Location of Qorveh county in Kurdistan province and Iran.

Kurdistan province is located in western Iran between 34°44' and 36°30' north latitude and 45°31' and 48°16' east longitude. Approximately, 1.71 percent of the whole country surface area is confined by this province where around 2 percent of Iranian population resides. Around half of the provincial population lives in rural areas and the most rural population is allocated to Qorveh County. Being the second greatest county of the province, Qorveh is located in southern Kurdistan (Figure 1).

Iran is divided into seven geological zones of sanandaj-sirjan, central Iran, loot, nehbandan, makran, kopedaq and alborz. The major part of the province belongs to the terminal part of Sanandaj-Sirjan zone while a small part lies within the Zagros folded belt. The Zagros part is mainly composed of thick red radiolarite and Biston limestone with Triassic to Upper Cretaceous age. Sanandaj-Sirjan zone, as the main one within the study area, is an intercontinental rift zone in which thick sequence of volcano-sedimentary rocks are accumulated. The study of metamorphism and deformation shows that this zone is one of the most dynamic parts of Iranian territory. Tectonostratigraphic units of this zone are of thick platform type deposit accumulated in an unstable continental edge. Therefore, most of the Paleozoic sequences reveal to the turbiditic type accumulated in troughs or trenches. Mesozoic rocks are of flysch type sediments associated in Mesozoic deep basin. These rocks are metamorphosed, deformed and intruded by several intrusive bodies. Tertiary rocks are scarce. It seems that this zone has been uplifted during Tertiary and the sea has been regressed. The study area like other parts of Sanandaj-Sirjan zone has an imbricate structure, which resulted into nappe stacking and crustal thickening. A group of volcanoes which were active within the upper Miocene and Pleistocene are located alongside the eastern frontiers of the county where at the moment some travertine springs are observed [22].

## Methods

**Figure 2:**
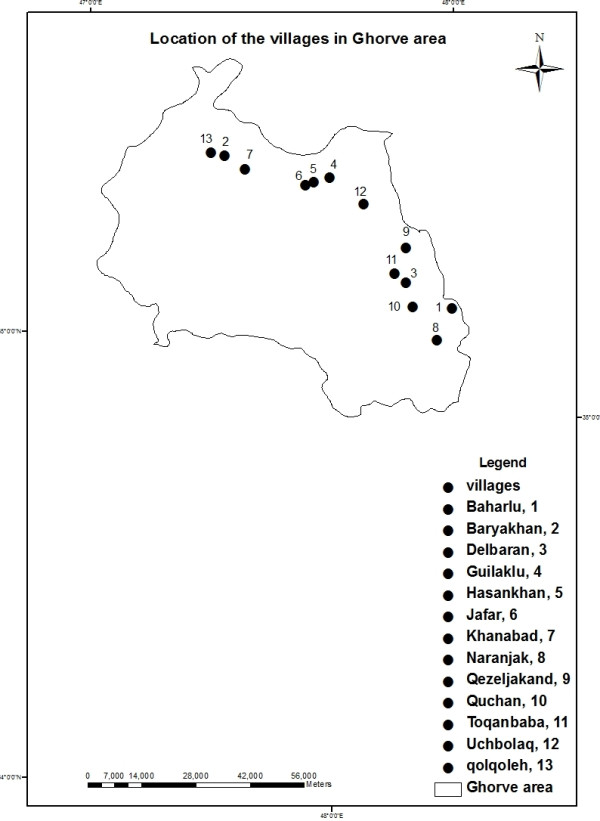
Groundwater sampling stations in Qorveh villages.

In order to select the most susceptible groundwater sampling stations in case of Arsenic concentration within the county, former studies [10,19,22,25] were regarded. Thirteen villages of Uchbolaq, Naranjak, Jafar, Hasankhan, Baharlu, Baryakhan, Khanabad, Qolqoleh, Toqanbaba, Qezeljakand, Guilaklu, Delbaran and Quchan in the County of Qorveh were taken into consideration to be monitored for the concentration of total arsenic in groundwater. The layout of thirteen boreholes is illustrated in Figure 2.

Composite sampling was considered for the study and through which five samples were collected from each station in August 2010. Sampling campaign was performed using a BAT Groundwater Sampler. Samples were collected in separate polyethylene bottles kept within 1:1 HNO3–H2O overnight and rinsed with distilled water. To prevent losses due to adsorption during analysis by the Silver Diethyldithiocarbamate (SDDC) method, the water samples were acidified with HCl to pH 2. For waters containing arsenic concentration values more than about 0.01 mg/L, The SDDC colorimetric method seems to be fairly reliable, cheap and rapid [25]. Arsenic in the sample is reduced to arsine, AsH3, in acid solution in a hydrogen generator. The arsine is passed through a scrubber to remove sulfide and is absorbed in a solution of silver diethyldithiocarbamate dissolved in pyridine. The red complex thus formed is measured in a spectrophotometer at 535 nm [26].

Accuracy and precision of the method was checked during the analytical procedure using synthetic and duplicate water samples according to Standard Methods. The quality control performed included a daily analysis of a standard and replicate analysis of samples and blanks. The satisfactory recovery rates for As was 92.4–105.3%.

Equations 1 and 2 are used to calculate the chronic daily intakes (CDI) for non-carcinogenic cases regarding to adults and children, respectively:

(1) $$\begin{array}{c} {\mathrm{CDI}}_{\mathrm{water-nc}-\mathrm{ing}}=\left[C_{\mathrm{g-water}}\times {\mathrm{EF}}_{\mathrm{resw}}\times {\mathrm{ED}}_{\mathrm{resw}}\times {\mathrm{IRW}}_{\mathrm{resw}}\right]\\ \;÷\left[365\times {\mathrm{ED}}_{\mathrm{resw}}\times {\mathrm{BW}}_{\mathrm{reswa}}\right] \end{array}$$

(2) $$\begin{array}{c} {\mathrm{CDI}}_{\mathrm{water-nc}-\mathrm{ing}}=\left[C_{\mathrm{g-water}}\times {\mathrm{EF}}_{\mathrm{reswc}}\times {\mathrm{ED}}_{\mathrm{reswc}}\times {\mathrm{IRW}}_{\mathrm{reswc}}\right]\\ \;÷\left[365\times {\mathrm{ED}}_{\mathrm{reswc}}{\times \mathrm{BW}}_{\mathrm{reswc}}\right] \end{array}$$

While equations 3 and 4 calculate the age-adjusted CDI for carcinogenic approach:

(3) $${\mathrm{CDI}}_{\mathrm{water-ca}-\mathrm{ing}}=\left[C_{\mathrm{g-water}}{\times \mathrm{IFW}}_{\mathrm{resw-adj}}\right]/{\mathrm{AT}}_{\mathrm{reswc}}$$

Where:

(4) $$\begin{array}{c} {\mathrm{IFW}}_{\mathrm{resw-adj}}=\left[{\mathrm{ED}}_{\mathrm{reswc}}{\times \mathrm{EF}}_{\mathrm{reswc}}{\times \mathrm{IRW}}_{\mathrm{reswc}}/{\mathrm{BW}}_{\mathrm{reswc}}\right]\\ \;+\left[\left[{\mathrm{ED}}_{\mathrm{resw}}{-\mathrm{ED}}_{\mathrm{reswc}}\right]{\times \mathrm{IRW}}_{\mathrm{reswa}}\right]/{\mathrm{BW}}_{\mathrm{reswa}} \end{array}$$

The excess lifetime cancer risk (ELCR) and the relevant hazard quotient (HQ) is estimated through equations 5 and 6, respectively:

(5) $$\mathrm{ELCR}={\mathrm{CDI}}_{\mathrm{water-ca}-\mathrm{ing}}{\times \mathrm{SF}}_{\mathrm{oral}}$$

(6) $$\mathrm{HQ}={\mathrm{CDI}}_{\mathrm{water-nc}-\mathrm{ing}}/\mathrm{RfD}$$

The input parameters for exposure assessment, risk and hazard analysis are indicated in Table 1.

**Table 1:** **Details for exposure assessment, risk and hazard analysis **[27]

**Parameter**	**Abbreviation**	**Unit**	**Value**
Chronic daily intake (water-non-carcinogenic-ingestion)	CDI _water-nc-ing_	mg/kg-day	---
Concentration	C_g-water_	mg/L	---
adjusted intake factor	IFW_resw-adj_	L/kg	380
Average time- noncarcinogenic	AT_resw_	day	10950
exposure duration - adult	ED_reswa_	year	30
exposure frequency - child	EF_reswc_	day/year	350
water intake rate - child	IRW_reswc_	L/day	1
body weight - child	BW_reswc_	kg	15
exposure duration - resident	ED_resw_	year	30
exposure duration - child	ED_reswc_	year	6
exposure frequency	EF_reswa_	day/year	350
water intake rate - adult	IRW_reswa_	L/day	2
body weight - adult	BW_reswa_	kg	70
Average time- carcinogenic	AT_reswc_	day	25550
Chronic daily intake (water-carcinogenic-ingestion)	CDI_water-ca-ing_	mg/kg-day	---
Oral slope factor	SF_oral_	(mg/kg-day)^-1^	1.5
Reference dose	RfD	mg/kg-day	3.00E-04

## Results and discussion

**Figure 3:**
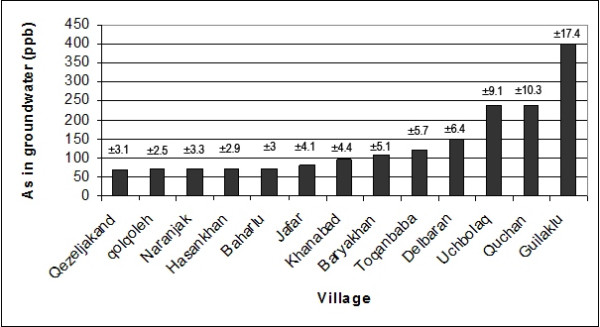
Concentration of arsenic in groundwater of studied villages.

The concentration of total arsenic in groundwater samples from different villages was analyzed. The results are indicated in Figure 3. As it is seen the highest arsenic concentration values belong to three villages of Guilaklu, Quchan and Uchbolaq while other ten villages show smaller values.

Exposure assessments on villagers were run according to the measured concentration values of arsenic in groundwater samples. The assessment was performed considering oral ingestion as the exclusive exposure route. Regarding carcinogenic and non-carcinogenic effects of arsenic, the exposure assessment results were used to calculate both potential risk and hazard.

Making use of the arsenic concentration in groundwater samples, the excess lifetime cancer risks through oral ingestion route is estimated by equation 4. Furthermore, to evaluate the non-carcinogenic threats caused by arsenic poisoning in drinking water, the non-carcinogenic chronic daily intake has been compared with arsenic oral reference dose (equation 5).

The more the value of HQ, the more the severity of the non-carcinogenic threats due to arsenic poisoning is observed within the area. Regarding the extremely higher significance of ingestion in comparison with other exposure routes for arsenic, ingestion hazard quotient has been widely used to indicate the relevant non-carcinogenic threats [23,28,29]. Quantitative risks and hazards due to arsenic poisoning in different villages are shown in Table 2.

**Table 2:** Arsenic concentration and relevant risk and hazard levels in different studied villages

**No.**	**Village**	**Arsenic concentration (μg/L)**	**Child Ingestion Hazard Quotient(HQ)**	**Adult Ingestion Hazard Quotient(HQ)**	**Excess lifetime cancer risk (ELCR)**
1	Uchbolaq	*240 ±9.1*	*4.92E+01 - 5.31E+01*	*2.11E+01 - 2.27E+01*	*5.15E-03 - 5.56E-03*
2	Naranjak	*72 ±3.3*	*1.46E+01 - 1.60E+01*	*6.27E+00 - 6.88E+00*	*1.53E-03 - 1.68E-03*
3	Jafar	*82 ±4.1*	*1.66E+01 - 1.83E+01*	*7.11E+00 - 7.86E+00*	*1.74E-03 - 1.92E-03*
4	Hasankhan	*72 ±2.9*	*1.47E+01 - 1.60E+01*	*6.31E+00 - 6.84E+00*	*1.54E-03 - 1.67E-03*
5	Baharlu	*72 ±3*	*1.47E+01 - 1.60E+01*	*6.30E+00 - 6.85E+00*	*1.54E-03 - 1.67E-03*
6	Baryakhan	*110 ±5.1*	*2.24E+01 - 2.45E+01*	*9.58E+00 - 1.05E+01*	2.34E-03 - *2.57E-03*
7	Khanabad	*96 ±4.4*	*1.95E+01 - 2.14E+01*	*8.37E+00 - 9.17E+00*	*2.04E-03 - 2.24E-03*
8	Qolqoleh	*70 ±2.5*	*1.44E+01 - 1.54E+01*	*6.16E+00 - 6.62E+00*	*1.51E-03 - 1.62E-03*
9	Toqanbaba	*120 ±5.7*	*2.44E+01 - 2.68E+01*	*1.04E+01 - 1.15E+01*	*2.55E-03 - 2.80E-03*
10	Qezeljakand	*67 ±3.1*	*1.36E+01 - 1.49E+01*	*5.84E+00 - 6.40E+00*	*1.43E-03 - 1.56E-03*
11	Guilaklu	*400 ±17.4*	*8.15E+01 - 8.89E+01*	*3.49E+01 - 3.81E+01*	*8.54E-03 - 9.31E-03*
12	Delbaran	*150 ±6.4*	*3.06E+01 - 3.33E+01*	*1.31E+01 - 1.43E+01*	*3.20E-03 - 3.49E-03*
13	Quchan	*240 ±10.3*	*4.89E+01 - 5.33E+01*	*2.10E+01 - 2.29E+01*	*5.12E-03 - 5.58E-03*

The highest and lowest arsenic concentrations are reported in Guilaklu and Qezeljakand villages with 420 and 67 μg/L, respectively. Such amounts seems to be extremely high when compared with the maximum concentration of Arsenic in groundwater throughout the Lanyang plain of northeastern Taiwan by 70.32 μg/L [30], Xiangjiang watershed, central-south China by 21.2 μg/L [31] as well as Kampong Cham and Kratie provinces in Cambodia by 2.37 and 140.60 μg/L, respectively [23].

**Figure 4:**
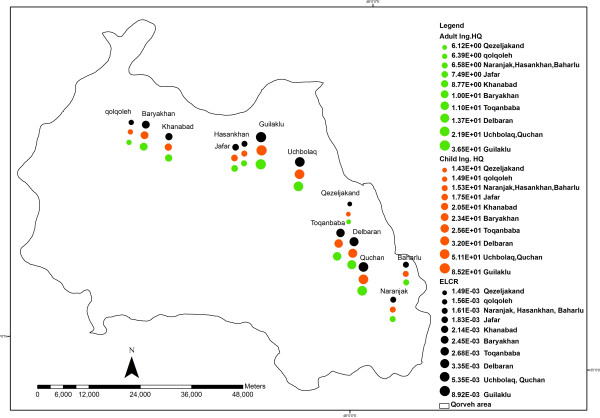
Spatial distribution of the hazard and ELCR due to arsenic poisoning in groundwater of Qorveh County.

The spatial distribution of age-adjusted ELCR and hazard levels among thirteen villages of the study area is illustrated in Figure 4.

As it is seen, the arsenic concentration in all water samples is higher than that allowed by US EPA and Institute of Standards and Industrial Research of Iran (ISIRI 1053) as the maximum contaminant level (10 ppb).

The highest arsenic concentration and consequently ELCR and hazard values belong to five villages of Guilaklu, Quchan, Uchbolaq, Delbaran and Toqanbaba situated alongside the eastern frontiers of Qorveh county.

## Conclusions

Spatial distribution of quantitative risk and hazard levels due to arsenic poisoning in groundwaters of thirteen villages in Qorveh County, Kurdistan province in western Iran is considered in this study. Existence of volcanic activities within the upper Miocene and Pleistocene in this part of the study area may be addressed as the main geopogenic source of arsenic pollution alongside the eastern frontiers of Qorveh county where villages like Guilaklu, Quchan, Uchbolaq, Delbaran and Toqanbaba are located.

HQs greater than 1 indicate a potential for an adverse effect to occur. All calculated HQ values are greater than 1 and 53.8% of the cases show values even greater than 10. In comparison with similar case studies in Izmir province, Turkey [28], Khsarch Andaet commune in Kratie province, Cambodia [23] and Kohistan region, northern Pakistan [24] by 19, 13.48 and 0% of HQ values greater than 1 respectively, a great potential for adverse effects threats the exposed habitants as well.

Risk values greater than one in a million (10^-6^) are generally considered unacceptable by the USEPA. However, this acceptable level may change according to national standards and environmental policies and may be as high as 10^-4^[32]. Quantitative excess lifetime cancer risk values are varying from 1.49E-03 in Qezeljakand to 8.92E-03 in Guilaklu. In this study, all exposed individuals have Excess lifetime cancer risk > 10^-3^, while 23% of the cases would experience an ELCR even greater than 0.005. This striking result shows that the whole population is at high-risk, even if only drinking water ingestion pathway is taken into consideration.

Risk level interpretation may be considered as even very high like the case in Kandal province groundwater within the Mekong River basin, Cambodia [23] where more than 92.5% of total cases had an ELCR value > 10^-3^. Additionally, it is important to keep in mind that this cancer assessment is estimated by considering ingestion as the sole exposure route; yet, the villagers are exposed to inhalation and dermal exposure routes as well every day.

Such drinking water source has the potential to impose adverse carcinogenic and non-carcinogenic effects on villagers. Accordingly, an urgent decision must be made to substitute the current drinking water source with a safer one. For regions like western Iran and southeastern Asian countries, online monitoring of arsenic levels in drinking water sources particularly in rural areas where no sophisticated water treatment facility is found, seems to be essential for the maintenance of public health [21].

In order to make an appropriate infrastructure for hygiene and health departments to implement preventive strategies for local and regional arsenic-related threats, having access to Geographic Information System (GIS) maps of arsenic contamination seems to be essential [25]. This study may be considered as a pioneer one to fulfill such commitment in western Iran.

## Abbreviations

(CDI): Chronic daily intakes; (ELCR): Excess lifetime cancer risk; (GIS): Geographic Information System; (HQ): Hazard quotient; (ISIRI): Institute of Standards and Industrial Research of Iran; (SDDC): Silver Diethyldithiocarbamate; (SMR): Standard mortality ratios

## Competing interests

Both authors declare that they have no competing interest.

## Authors’ contributions

TN participated in site selection, arsenic concentration analysis and risk and hazard analysis. NSB participated in map generation and data analysis. Both authors read and approved the final manuscript.

## Authors’ information

Touraj Nasrabadi, PhD in environmental engineering, Assistant professor, Graduate Faculty of Environment, University of Tehran.

Niloufar Shirani bidabadi, M.Sc. Student of environmental planning, management and education, Graduate Faculty of Environment, University of Tehran.
